# Detection of hydrogen peroxide in Photosystem II (PSII) using catalytic amperometric biosensor

**DOI:** 10.3389/fpls.2015.00862

**Published:** 2015-10-15

**Authors:** Ankush Prasad, Aditya Kumar, Makoto Suzuki, Hiroyuki Kikuchi, Tomoya Sugai, Masaki Kobayashi, Pavel Pospíšil, Mika Tada, Shigenobu Kasai

**Affiliations:** ^1^Biomedical Engineering Research Center, Tohoku Institute of TechnologySendai, Japan; ^2^Department of Biophysics, Faculty of Science, Centre of the Region Haná for Biotechnological and Agricultural Research, Palacký UniversityOlomouc, Czech Republic; ^3^Graduate Department of Environmental Information Engineering, Tohoku Institute of TechnologySendai, Japan; ^4^Graduate Department of Electronics, Tohoku Institute of TechnologySendai, Japan; ^5^Center for General Education, Tohoku Institute of TechnologySendai, Japan

**Keywords:** photosystem II, superoxide anion radical, hydrogen peroxide, reactive oxygen species, amperometric biosensor, EPR-spin trapping

## Abstract

Hydrogen peroxide (H_2_O_2_) is known to be generated in Photosystem II (PSII) via enzymatic and non-enzymatic pathways. Detection of H_2_O_2_ by different spectroscopic techniques has been explored, however its sensitive detection has always been a challenge in photosynthetic research. During the recent past, fluorescence probes such as Amplex Red (AR) has been used but is known to either lack specificity or limitation with respect to the minimum detection limit of H_2_O_2_. We have employed an electrochemical biosensor for real time monitoring of H_2_O_2_ generation at the level of sub-cellular organelles. The electrochemical biosensor comprises of counter electrode and working electrodes. The counter electrode is a platinum plate, while the working electrode is a mediator based catalytic amperometric biosensor device developed by the coating of a carbon electrode with osmium-horseradish peroxidase which acts as H_2_O_2_ detection sensor. In the current study, generation and kinetic behavior of H_2_O_2_ in PSII membranes have been studied under light illumination. Electrochemical detection of H_2_O_2_ using the catalytic amperometric biosensor device is claimed to serve as a promising technique for detection of H_2_O_2_ in photosynthetic cells and subcellular structures including PSII or thylakoid membranes. It can also provide a precise information on qualitative determination of H_2_O_2_ and thus can be widely used in photosynthetic research.

## Introduction

Photosystem II (PSII) is a multi-subunit pigment-protein complex which is located in the thylakoid membrane of chloroplasts of cyanobacteria, algae and higher plants that comprises of more than 25 proteins and the concomitant cofactors (Ferreira et al., 2004; Loll et al., 2005; Guskov et al., 2009; Kawakami et al., 2011). In plants, photosynthesis has been considered as a source of reactive oxygen species (ROS) production which works in close association with regulated mechanism of antioxidant network under normal conditions. The ROS in plants are known to be involved in cell toxicity, defense and signaling, and have been recently overviewed (Krieger-Liszkay, 2005; Foyer and Shigeoka, 2011; Schmitt et al., 2014).

Chlorophyll pigments of the PSII antenna complex absorb light energy and use it for the oxidation of water molecules and reduction of plastoquinone. Light energy absorbed by chlorophyll pigments converted into the energy of separated charges and consequent water-plastoquinone oxidoreductase activity is involuntarily linked with the production of ROS (Pospíšil, 2009, 2012). In unison, released molecular oxygen serves as a forerunner of ROS, which at low concentration play an important role in cell regulation, whereas if formed in excess, is responsible for oxidation of biomolecules such as lipid, proteins, and nucleic acid (Halliwell and Gutteridge, 2007). In addition, direct oxidation of proteins and lipids by UV irradiation and toxic chemicals following subsequent chemical reactions are also known to be associated with formation of ROS (Halliwell and Gutteridge, 2007; Prasad and Pospíšil, 2012).

Production of ROS arises when excitation energy transfer to the PSII reaction center is inadequate or there is inhibition of electron transport chain in PSII. The redox couples in PSII covers a broad range of redox potential, it ranges from a very high negative value of redox couple, Pheo/Pheo^−^ (*E*m = −610 mV) to a very high positive value of redox couple P680^+^/P680 (*E*m = +1250 mV) (Rappaport and Diner, 2008; Pospíšil, 2012). PSII is capable of either oxidizing water molecule or reducing molecular oxygen on the electron donor and on the electron acceptor side of the membrane, respectively (Pospíšil, 2012). There is leakage of electrons to molecular oxygen during the electron transport on the electron acceptor side of PSII (Pospíšil, 2009).

Formation of O${}_{2}^{•-}$ results from non-enzymatic and enzymatic one-electron reductions of molecular oxygen. Pheophytin (Pheo^•−^) (Ananyev et al., 1994; Pospíšil et al., 2004), tightly bound plastosemiquinone (Q${}_{A}^{•-}$), (Cleland and Grace, 1999; Pospíšil et al., 2004), loosely bound plastosemiquinone (Q${}_{B}^{•-}$), (Zhang et al., 2003; Yadav et al., 2014), and free plastosemiquinone (PQ^•−^) (Mubarakshina and Ivanov, 2010) maintains non-enzymatic reduction of molecular oxygen to O${}_{2}^{•-}$. Heme iron of low-potential (LP) form of cyt *b*_559_ reduces molecular oxygen to O${}_{2}^{•-}$ in the enzymatic reaction pathway (Pospíšil et al., 2006; Pospišil, 2011).

One electron reduction of O${}_{2}^{•-}$ either via non-enzymatic or enzymatic reaction pathway results in the formation of H_2_O_2_. In spontaneous dismutation, O${}_{2}^{•-}$ provides an additional electron to another O${}_{2}^{•-}$, and then with protonation brings about the formation of H_2_O_2_. In enzymatic dismutation, the ferrous heme iron of HP form of cyt *b*_559_ drives the catalysis of one-electron reduction of HO${}_{2}^{•}$ to H_2_O_2_ (Tiwari and Pospísil, 2009; Pospišil, 2011). Spontaneous dismutation is preferred where there is availability of protons e.g., at the membrane edge while PSII metal centers are chosen to catalyze the dismutation reaction in the interior of the membrane (Pospíšil, 2012). The one-electron reduction of O${}_{2}^{•-}$ to H_2_O_2_ is catalyzed by superoxide dismutase (SOD) and is known to occur predominantly in the mitochondria, peroxisomes, and cytoplasm. At the physiological pH, the dismutation reaction is preferably catalyzed by SOD.

Several spectroscopic techniques (fluorescence and chemiluminescence) and chromatographic techniques (high performance liquid chromatography coupled with peroxyoxalate chemiluminescence detection) have been used in the past for the determination of H_2_O_2_ in living cells (Mills et al., 2007; Chen et al., 2009; Ahammad, 2013). Light induced production of H_2_O_2_ have been measured in PSII membranes by oxidation of thiobenzamide with lactoperoxidase. Thiobenzamide sulfoxide was quantified by its absorbance at 370 nm (Schröder and Åkerlund, 1990; Arató et al., 2004; Pospíšil et al., 2004). Production of H_2_O_2_ by chloroplasts have been measured by the AmplexRed fluorescence assays (Mubarakshina and Ivanov, 2010; Yadav and Pospíšil, 2012). Hydrogen peroxide (H_2_O_2_) detecting probes, 3,3 diaminobenzidine (DAB), Amplex Red (AR), Amplex Ultra Red (AUR), and a europium-tetracycline complex (Eu3Tc) have been compared by infiltrating into tobacco leaves and tested for sensitivity to light, toxicity, subcellular localization, and capacity to detect H_2_O_2_ *in vivo* (Šnyrychová et al., 2009). The induction of H_2_O_2_ generation at the leaf level after 3-acetyl-4-hydroxyl-5-isopropylpyrroline-2-dione (3-AIPTA) or bentazon treatment has been detected by performing histochemical analysis with 3, 3 DAB staining (Chen et al., 2012). Apart from several spectroscopic techniques (absorbance, fluorescence, chemiluminescence), cyclic voltammetry and histochemical technique, a reporter system based on HSP70A promoter-luciferase fusions have also been developed in past for the detection of H_2_O_2_ *in vivo* (Shao et al., 2007).

**Scheme 1:**
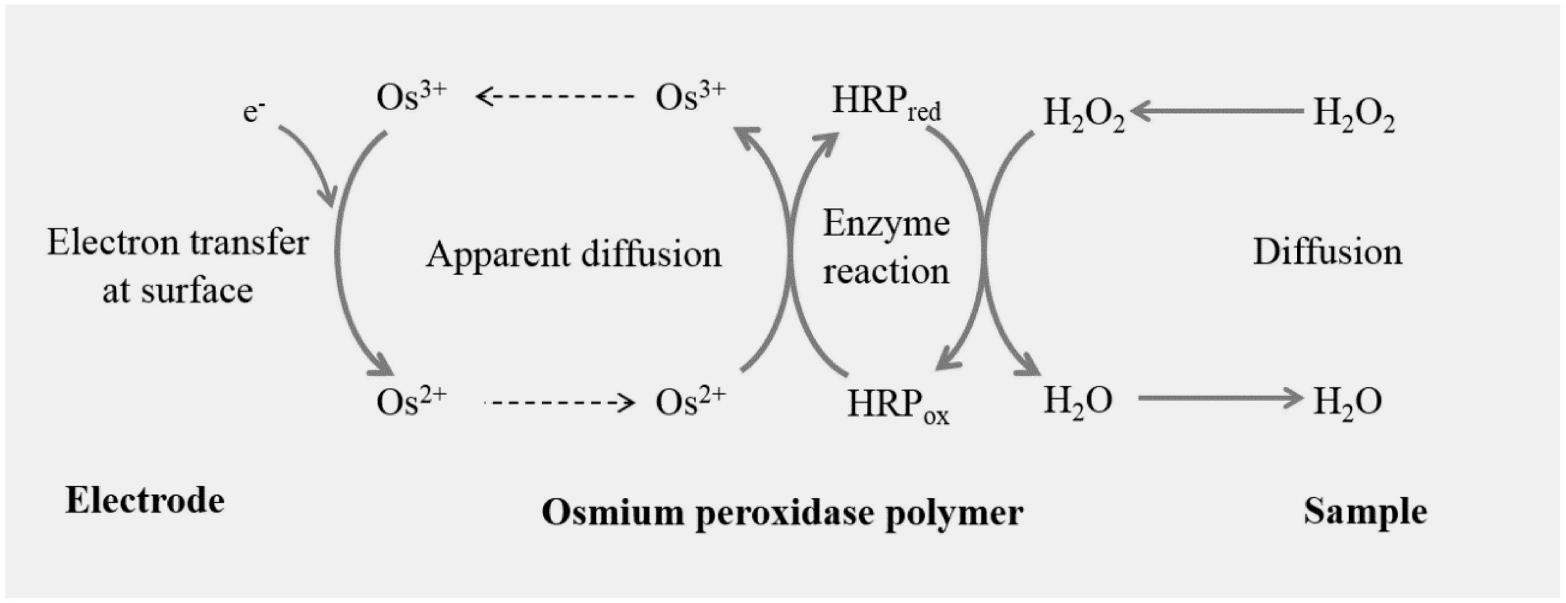
**Working principle of catalytic amperometric biosensor device: schematic illustration shows the working principle of Osmium-horseradish peroxidase (Os-HRP) modified carbon electrode depicting the oxidation-reduction cycle leading to generation of reduction current for H_2_O_2_**.

In the electrochemical method, mediator and non-mediator based electrode have been used in the past. Among the mediator based modified electrodes, HRP is the most commonly used material for the modification of the electrode in the last decade (Lin et al., 2000; Camacho et al., 2007; Radi et al., 2009; Ahammad, 2013). The sensitivity and selectivity of H_2_O_2_ biosensor depends on the material used and modifications involved (Lin et al., 2000; Nakabayashi and Yoshikawa, 2000; Yao et al., 2005; Wang and Zhang, 2006; Camacho et al., 2007; Radi et al., 2009; Inoue et al., 2010; Li et al., 2013). The mechanism of detection of H_2_O_2_ biosensor is described in Scheme 1. The enzyme HRP is converted to its oxidized form, which is than reduced at the surface of the carbon electrode by the transfer of the electron via the mediator. Different mediators have been used in the past including methylene blue (Lin et al., 2000; Kafi et al., 2009; Tiwari and Singh, 2011); quinones predominantly hydroquinones (Lei et al., 2004, 2005; Zhang et al., 2008; Yang et al., 2010); ferrocene (Wang et al., 2005; Senel et al., 2010), and ferrocene carboxylic acid (Tian et al., 2001; Tripathi et al., 2006; Liu et al., 2011; Luo et al., 2011). The detection limit in utmost cases were found to be in the concentration range of units of millimolar or micromolar (μM) while a limited reports showed detection limit in tens of nanomolar (nM) concentration (Zhang et al., 2008; Loew et al., 2009; Lu et al., 2010). Recently, Os-HRP was compared with native HRP based coated glassy carbon electrode and it was found to possess high sensitivity for hydroperoxide. The electrodes were tested for H_2_O_2_ and hydroperoxide in the concentration range of 0.01–1 μM (Loew et al., 2009).

Non-mediator based H_2_O_2_ biosensor has also been widely used in the past; however, it is known that the electron transfer between HRP and the electrode is difficult due to higher distance between the active site of HRP and the electrode. The voltammetric detection of H_2_O_2_ at carbon electrodes is challenging due to the slow electron transfer kinetics associated with the irreversible oxidation of peroxide. An anodic scan has been used as an electrochemical pretreatment and a rapid, sensitive and selective voltammetric method has been developed for the detection of physiological concentrations of H_2_O_2_ at uncoated carbon fiber microelectrodes (Sanford et al., 2011).

In this study, we provide an experimental evidence on the detection of H_2_O_2_ by using Osmium (Os) as a mediator which promotes shuttling of electrons between the electrode and the enzyme. Detection of H_2_O_2_ by using highly sensitive and selective Os-HRP modified electrode was tested in PSII membrane under light illumination. The current study introduces the use of catalytic amperometric biosensors in detection of low level of H_2_O_2_ production in PSII membrane.

## Materials and methods

### Material and chemical reagents

5-(ethoxycarbonyl)-5-methyl-1-pyrroline N-oxide (EMPO) spin trap was obtained from Alexis Biochemicals (Lausen, Switzerland). Capillary tube used for Electron paramagnetic resonance (EPR) measurements was purchased from Blaubrand intraMARK, Brand, Germany. All other chemicals of analytical grade were purchased either from Wako Pure Chemicals Industries, Ltd. (Osaka, Japan), Sigma-Aldrich chemie Gmbh (Munich, Germany), or Sigma-Aldrich Japan K.K (Tokyo, Japan).

### Preparation of PSII membrane

Photosystem II (PSII) membranes were prepared from fresh spinach leaves using the method reported previously by Berthold et al. (1981) with modifications described by Ford and Evans (1983). All steps during the isolation procedure were done at 4°C in green light using green LED strip (Photon Systems Instruments (PSI), Drásov, Czech Republic) or under dark condition using different buffers (A and B). The composition of buffer A (pH 7.5) being 400 mM sucrose, 15 mM NaCl, 5 mM MgCl_2_, 5 mM CaCl_2_, 40 mM HEPES (pH 7.5), 5 mM Na-ascorbate, and 2 g/l bovine serum albumin (BSA) while buffer B was composed of 400 mM sucrose, 15 mM NaCl and 5 mM MgCl_2_, 40 mM MES (pH 6.5). Spinach leaves were washed twice with deionized water. Na-ascorbate and BSA were added immediately before crushing the spinach leaves. Dark adapted leaves (400 g) were homogenized with 500 ml of buffer A. This step was followed by filtering the homogenized mixture through 2 layers of nylon bolting cloth. Filtrate was transferred into ice-chilled centrifugation tubes and was centrifuged for 10 min at 9950 × g at 4°C. The supernatant was thrown out and pellet was mixed properly with paint brush. The pellet was then resuspended in 600 ml of buffer B and again centrifuged at 9950 × g for 10 min at 4°C. After the centrifugation, supernatant was discarded and pellet was again resuspended in buffer B, at this step concentration of chlorophyll was measured. This step was followed by treating the suspension with 5% Triton X-100 on ice bath with continuous stirring for 17 min, and then centrifugation at 7000 × g for 7 min. The pellet was discarded and supernatant was centrifuged again at 48,000 × g for 20 min at 4°C. Pellet was washed for 3 times with buffer B and at the final step, and chlorophyll concentration was measured. PSII membrane were diluted to final chlorophyll concentration (3–6 mg Chl ml^−1^) and were stored at −80°C until further use.

Chlorophyll concentration was determined in aqueous 80 % (v/v) acetone by absorbance at 646 and 663 nm according to the method described by Lichtenthaler (1987).

### Light illumination

PSII membranes were exposed to continuous white light (1,000 μmol photons m^−2^s^−1^) for time period as required during the different experimental setups. The illumination was performed using halogen lamps with a light guide (KL1500 Electronic, Schott, Mainz, Germany and PL075, Hoya Candeo Optonics, Japan). The light intensity was measured by quantum radiometer LI-189 and LI-185B (LI-COR Inc., Lincoln, U.S.A.).

### Electron paramagnetic resonance (EPR) spin-trapping spectroscopy

The detection of O${}_{2}^{•-}$ was performed by EPR spin-trapping spectroscopy. Superoxide anion radical was detected by spin trapping using EMPO, 5-(ethoxycarbonyl)-5-methyl-1-pyrroline N-oxide. The experimental conditions are as follows: PSII membranes (150 μg Chl ml^−1^); EMPO, 25 mM; phosphate buffer, 40 mM (pH 6.5), DCMU, 20 μM and desferal, 50 μM. Spectra were recorded using EPR spectrometer Mini Scope MS400 (Magnettech GmbH, Berlin, Germany) with EPR conditions as follows: microwave power, 10 mW; modulation amplitude, 1 G; modulation frequency, 100 kHz; sweep width, 100 G; scan rate, 1.62 G s^−1^. Formation of light-induced EMPO-OOH adduct EPR spectra was measured in PSII membranes after illumination of PSII membranes in glass capillary tube with continuous white light (1000 μmol photons m^−2^ s^−1^).

### Fabrication of Osmium-HRP (Os-HRP) modified carbon electrode

The electrochemical biosensor used in the study was prepared using carbon-electrode (BAS Inc, ALS Co., Ltd., Japan). Prior to each measurement, the carbon electrode was cleaned using PK-3 Electrode Polishing Kit (BAS Inc., ALS Co., Ltd., Japan) with the aim to remove redox reaction products accumulated on the electrode surface. This step was followed by spreading/dropping of 0.5 μL aliquot of Os-HRP polymer solution (Bioanalytical System, USA) on the carbon electrode (φ, 1 mm). The solution was allowed to form a circular thin film after overnight drying at 4°C under dark condition.

### Cyclic voltammetry of Os-HRP modified carbon electrode

For the basic characterization of the Os-HRP modified carbon electrode, a portion of the electrode was soaked in 10 ml of 40 mM Na-phosphate buffer and cyclic voltammetry was performed. Cyclic voltammetry was conducted at a scan rate of 20 mV/s from 0.0 to +0.5 V at room temperature.

### Hydrogen peroxide detection using catalytic amperometric biosensor

The electrochemical measurements were performed using a potentiostat (HA1010mM4S; Hokuto Denke Co. Ltd., Japan). For the assay to study H_2_O_2_ generated in PSII membrane, the PSII membranes at a concentration of 150 μg Chl ml^−1^ was introduced into a six well Repro plate (IFP, Research unit for the functional peptides, Yamagata, Japan). Ag/AgCl electrode was used as a reference electrode and Os-HRP modified carbon electrode was used as working electrode. Platinum plate in the dimension of 5^*^5^*^0.1 mm was used as a counter electrode. Kinetics on the formation of light-induced H_2_O_2_ was measured in PSII membranes after illumination under continuous white light (1000 μmol photons m^−2^ s^−1^) for a duration of 1 h. The sampling time was kept at 500 ms.

## Results

### Superoxide anion radical production in PSII membranes

**Figure 1:**
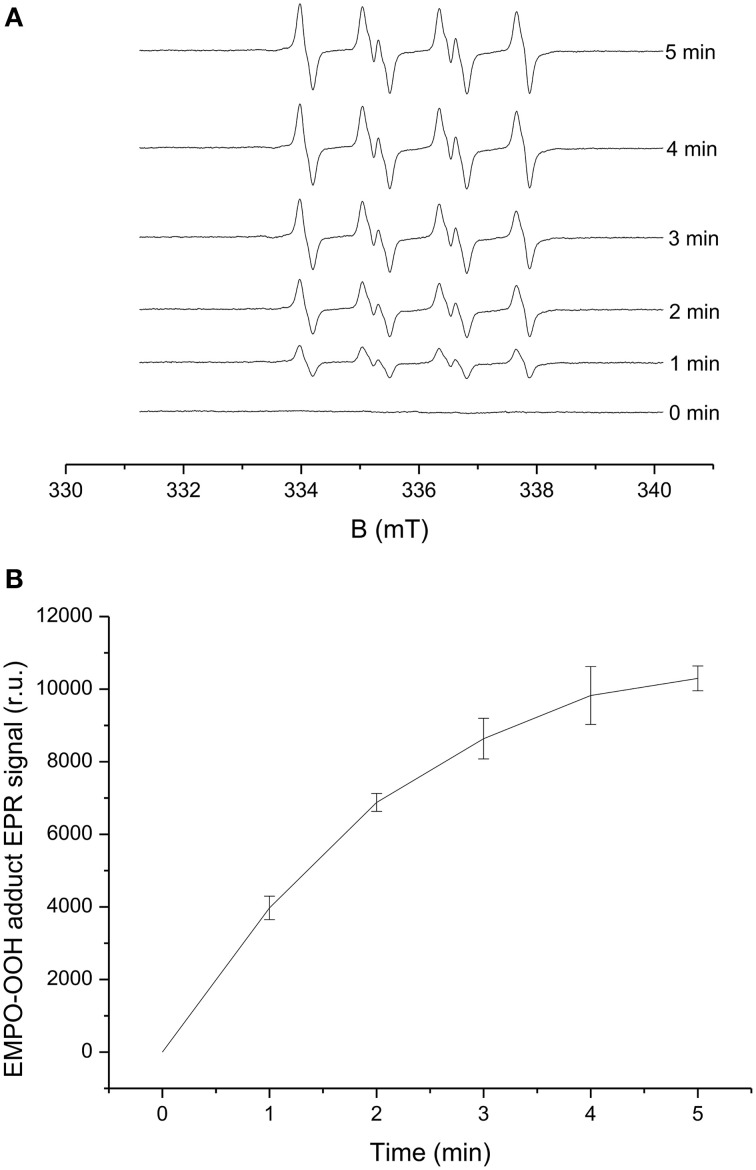
**Light-induced EMPO-OOH adduct EPR spectra measured in PSII membranes**. EMPO-OOH adduct EPR spectra were measured under light illumination (1000 μmol photons m^−2^ s^−1^) of PSII membranes (150 μg Chl ml^−1^) in the presence of 25 mM EMPO, 100 μM desferal, and 40 mM phosphate buffer (pH 6.5). **(A)** Shows the spectra measured in the time range of 0–5 min of illumination and 1 **(B)** shows time profile of EMPO-OOH adduct EPR spectra. The intensity of EPR signal was evaluated by measuring the relative height of central peak of the first derivative of the EPR absorption spectrum. The data represent the mean value (±SD) where *n* = 3.

The light-induced formation of O${}_{2}^{•-}$ in PSII membranes was measured using EPR spin-trapping spectroscopy. The spin-trapping was accomplished by the spin-trap compound 5-(ethoxycarbonyl)-5-methyl-1-pyrroline N-oxide (EMPO). The EMPO did not induce any EMPO-OOH adduct EPR signal in non-illuminated PSII membranes, whereas illumination with a continuous white light (1,000 μmol photons m^−2^ s^−1^) resulted in the formation of the EMPO-OOH adduct EPR signal (Figure 1A). Time dependence of EMPO-OOH adduct EPR signal shows that formation of O${}_{2}^{•-}$ is enhanced linearly for a duration upto 5 min of light illumination (Figure 1B). These results suggest that illumination of PSII membranes with a continuous white light (1,000 μmol photons m^−2^ s^−1^) results in O${}_{2}^{•-}$ production.

### Effect of superoxide dismutase and catalase on O${}_{2}^{•-}$ production in PSII membranes

**Figure 2:**
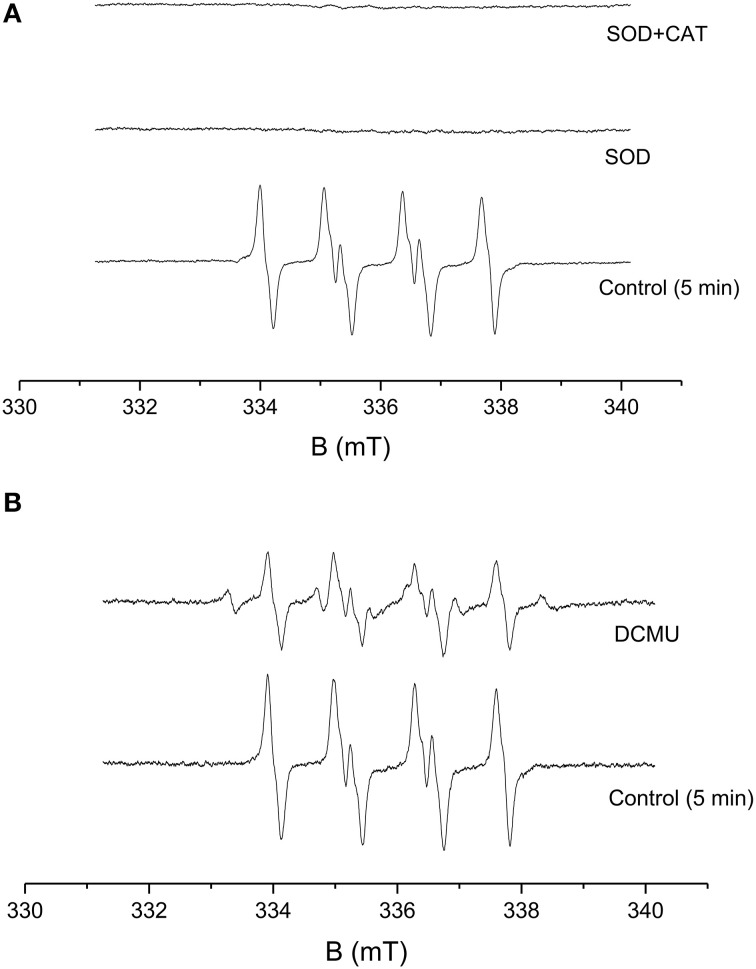
**Effect of SOD, CAT, and DCMU on EMPO-OOH adduct EPR spectra measured in PSII membranes**. EMPO-OOH adduct EPR spectra were measured in PSII membranes in the presence of SOD and CAT under light illumination. The relative intensity of the light-induced EMPO-OOH adduct EPR signal measured in the presence of SOD (400 U/ml) and SOD+CAT (400 U/ml each) **(A)** and DCMU (20 μM) **(B)**. The other experimental conditions were the same as described in Figure 1.

To study the contribution of O${}_{2}^{•-}$ leading to H_2_O_2_ formation in PSII membranes under light illumination, the effect of SOD and catalase (CAT) were studied on O${}_{2}^{•-}$. Upon addition of exogenous SOD, which is known to catalyze the dismutation of O${}_{2}^{•-}$ to H_2_O_2_ to PSII membranes prior to illumination, EMPO-OOH adduct EPR signal was found to diminish completely. The simultaneous addition of SOD and CAT was also found to suppress the EMPO-OOH EPR signal completely (Figure 2A). This observation indicates that O${}_{2}^{•-}$ produced during light illumination is most likely involved in H_2_O_2_ formation in PSII membranes.

### Effect of DCMU on O${}_{2}^{•-}$ production in PSII membranes

The effect of herbicides, DCMU [3-(3,4-dichlorophenyl)-1,1-dimethylurea] (Sigma Aldrich, Germany) was tested for its effect on EMPO-OOH adduct EPR signal in PSII membranes. The effect of DCMU which is known to block the electron transfer from Q_*A*_ to Q_*B*_ was studied on light-induced formation of O${}_{2}^{•-}$. Figure 2B shows that the addition of DCMU suppressed EMPO-OOH adduct EPR signal approximately by 50% (Figure 2B). These observations indicate that loosely bound plastosemiquinone bound at or after the Q_*B*_ site can contribute to the overall production of O${}_{2}^{•-}$ via the reduction of molecular oxygen.

### Characterization of Os-HRP modified carbon electrode

**Figure 3:**
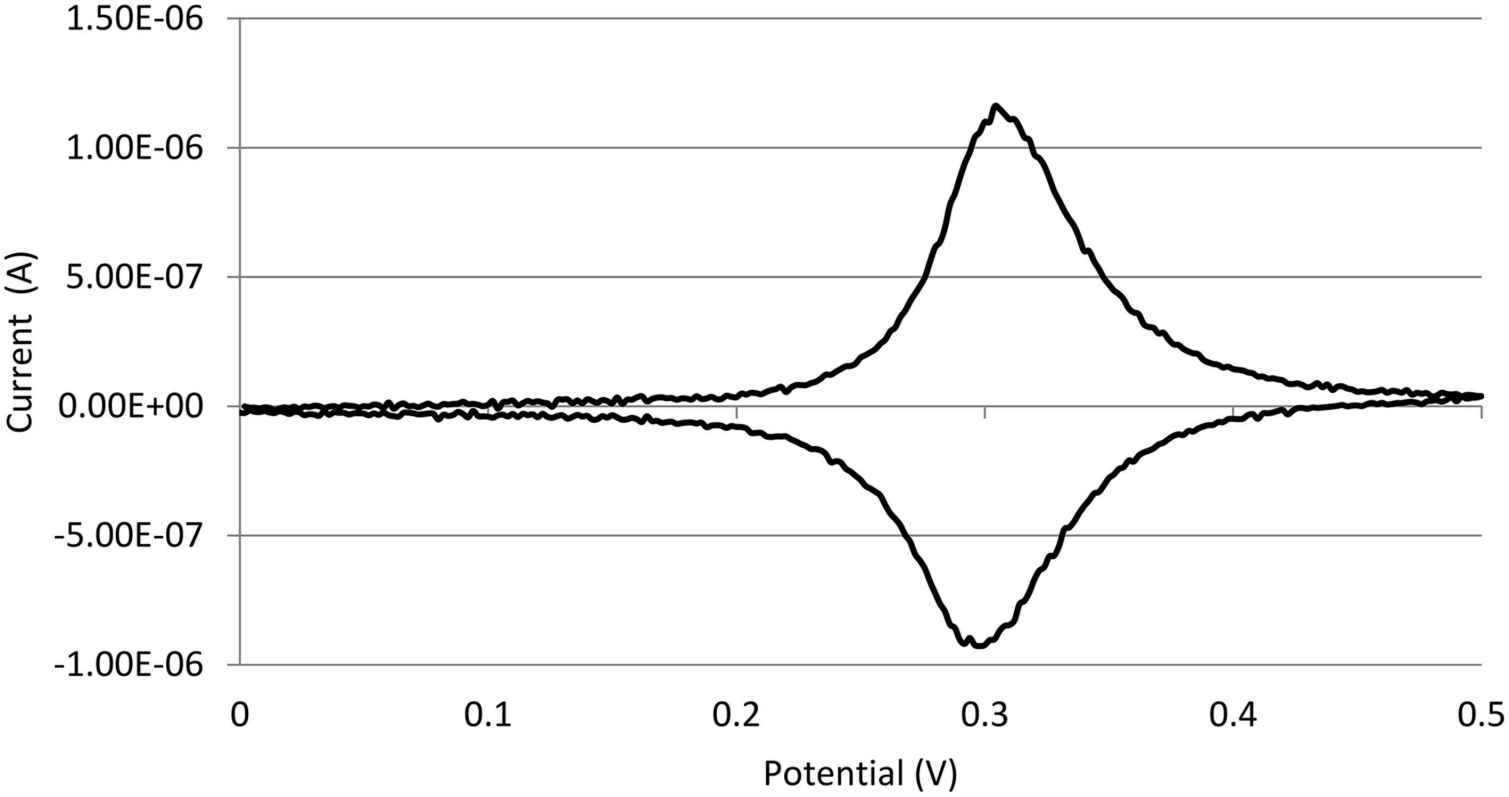
**Characterization of Os-HRP modified carbon electrode**. Characterization of the Os-HRP modified carbon electrode: cyclic voltammetry was performed for the basic characterization of the modified electrode. Cyclic voltammetry was conducted at a scan rate of 20 mV/s from 0.0 to +0.5 V at room temperature.

The characterization of the Os-HRP modified carbon electrode was performed using cyclic voltammetry (Figure 3). Cyclic voltammetry was conducted at a scan rate of 20 mV/s from 0.0 to + 0.5 V at room temperature. The oxidation and reduction current were obtained at 0.3 V vs. Ag/AgCl. Based on the data obtained, the surface concentration of Os-HRP on the carbon electrode was calculated to be 6.78 × 10^−9^ mol/cm^2^. The Calibration curve of Os-HRP modified carbon electrode was also measured using standard H_2_O_2_ solution (Supplementary Data).

### Hydrogen peroxide production in PSII membranes

**Figure 4:**
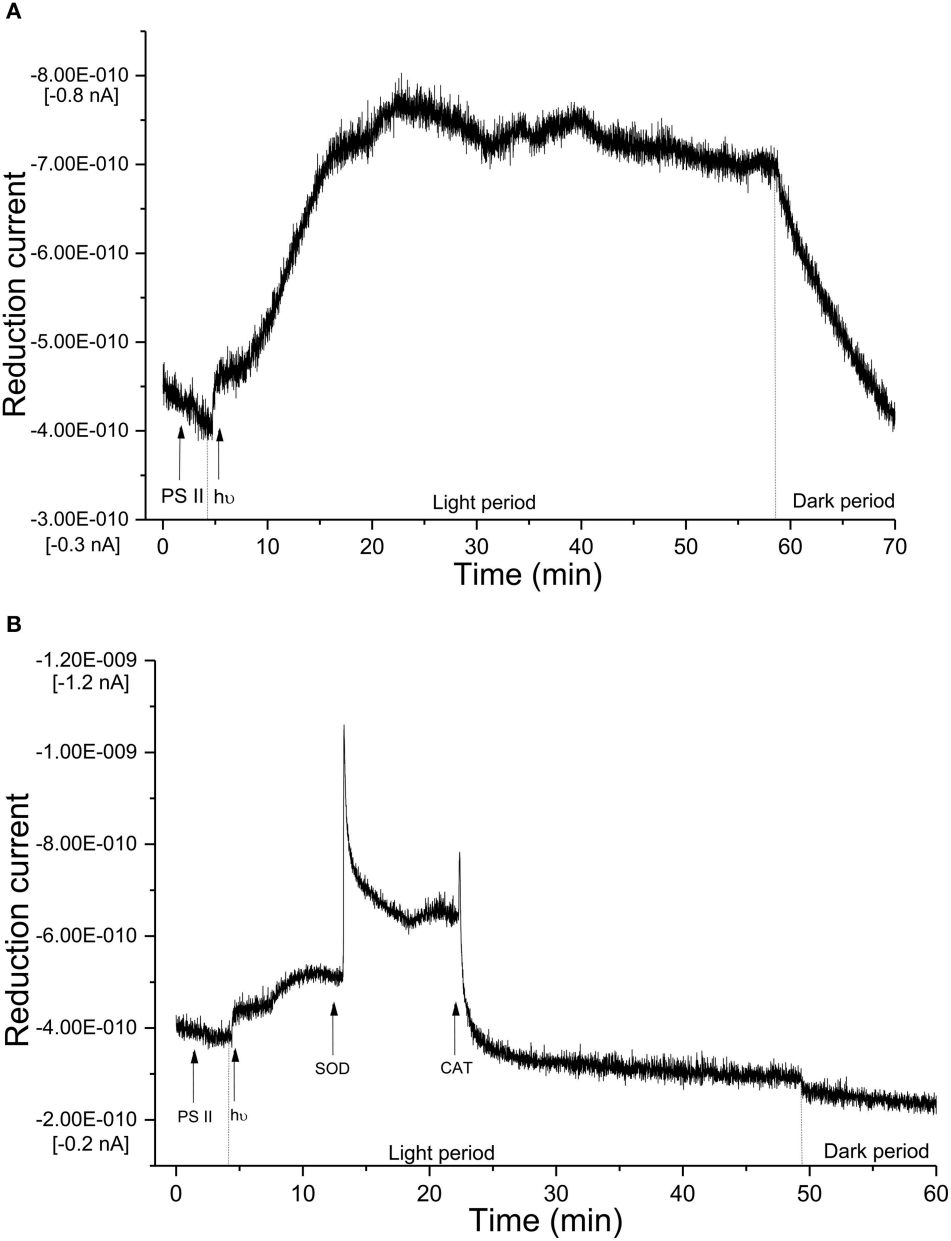
**Real-time monitoring of reduction current for hydrogen peroxide in PSII membrane. (A)** Kinetics of the production of H_2_O_2_ was measured using Os-HRP modified carbon electrode during light illumination in PSII membranes. The light illumination was started at 5 min from the start of the measurement and the reduction current was measured for a duration of 1 h. **(B)** Effect of SOD and CAT on reduction current was measured in the presence of SOD (400 U/ml) and SOD + CAT (400 U/ml each).

The light-induced formation of H_2_O_2_ in PSII membranes was measured using catalytic amperometric biosensor. The study of kinetics of H_2_O_2_ production was accomplished using Os-HRP modified carbon electrode. The reduction current generated in the presence of H_2_O_2_ was monitored. Under dark condition, no change in the reduction current was observed whereas illumination with a continuous white light (1,000 μ mol photons m^−2^ s^−1^) resulted in change in the reduction current. Figure 4A shows typical chronoamperometric responses, the reduction currents gradually increased after light illumination followed by a shoulder which continues for a duration of about 1 h which than rapidly drops at the switching off the light (dark period). The peak value of the reduction current was reached after about 15 min of light illumination with a maximum change in reduction currents of approximately 400 pA. The data presented shows continuous generation of H_2_O_2_ till the period of light illumination (Figure 4A). These results shows that illumination of PSII membranes with continuous white light results in H_2_O_2_ production.

### Effect of SOD and CAT on H_2_O_2_ production in PSII membranes

To monitor the functionality of the sensor, the effect of SOD was measured under light-illumination. Reduction current for H_2_O_2_was measured for first 5 min of light illumination where a linear increase was observed similar to control (Figure 4A). Upon addition of exogenous SOD to PSII membranes during illumination, a fast increase in reduction current was observed bringing a considerable change of about 600 pA. The fast increase was followed by a rapid drop however; the reduction current was still higher as compared to reduction current recorded before the addition of SOD. The addition of CAT completely suppressed the reduction current by about 100% (Figure 4B). These results show that under illumination of PSII membranes with a continuous white light, H_2_O_2_ production in PSII membrane is contributed via dismutation of O${}_{2}^{•-}$.

## Discussion

In the current study, we used spectroscopic and amperometric techniques to measure the production of ROS in spinach PSII membrane. Our prime objective was to study the effect of high light stress in PSII membrane reflected by ROS production primarily the generation of H_2_O_2_ as the stress response formed via the dismutation of O${}_{2}^{•-}$. Superoxide anion radical which is known to be formed by one-electron reduction of molecular oxygen was measured under light illumination (Figure 1). EPR spin-trapping data obtained using the urea-type herbicide (DCMU) supports the evidence on the involvement of loosely bound plastosemiquinone in O${}_{2}^{•-}$ production (Figure 2B). Suppression of EMPO-OOH adduct EPR signal is in agreement with our previously published results where contribution of loosely bound plastosemiquinone at or after the Q_*B*_ site (DCMU-sensitive site) might contribute to the overall O${}_{2}^{•-}$ production (Yadav et al., 2014). The EMPO-OOH adduct EPR signal in the presence of DCMU indicates that molecular oxygen is reduced prior to the Q_*B*_ site which is known to occur due to reduction of molecular oxygen by Pheo^•−^ or Q${}_{A}^{•-}$ which serves as electron donors to molecular oxygen due to their low redox potentials (Pospíšil, 2012). The observation that the EMPO-OOH adduct was completely suppressed in PSII membranes illuminated in presence of SOD indicates that O${}_{2}^{•-}$ formed during the light illumination dismutates to H_2_O_2_ prior to its interaction with spin trap (Figure 2A). It can be concluded here that the H_2_O_2_ formed in PSII membrane under light illumination is contributed by the dismutation of O${}_{2}^{•-}$.

Amperometric methods for the direct detection of H_2_O_2_ has been used since last decades in animal cells (Mouithys-Mickalad et al., 2001; Inoue et al., 2010) however, very limited evidences exist on its application in plant cells (Cleland and Grace, 1999). Amperometric measurements have been implemented at the level of protoplast to study the photosynthetic activity under the effect of benzoquinone (Yasukawa et al., 1998, 1999). Redox response of benzoquinone, p-hydroquinone and oxygen was measured by placing a microelectrode close to an algal protoplast to localize concentration of these species (Yasukawa et al., 1999). Direct detection of H_2_O_2_using electrochemical methods; however, had never been reported previously in photosynthetic organelles. H_2_O_2_ detection in subchloroplast oxygen-evolving PSII particles and isolated reaction center complexes of PSII has been studied using luminol-peroxidase chemiluminescence and pulse photoactivation (Zastrizhnaya et al., 1997). Production of H_2_O_2_ was detected in PSII membranes using AR fluorescent assay (Yadav and Pospíšil, 2012). Levels of H_2_O_2_ including O${}_{2}^{•-}$ and singlet oxygen has been determined by using both histochemical and fluorescent probes in leaves and thylakoids (Zulfugarov et al., 2014), however, there exist arguments over the selectivity of the molecular probes used.

Two different approaches of the detection of H_2_O_2_via electrochemical methods are being used in animal cells. The detection of H_2_O_2_ is either via a non-mediator or a mediator based biosensor device (Lin et al., 2000; Nakabayashi and Yoshikawa, 2000; Yao et al., 2005; Wang and Zhang, 2006; Camacho et al., 2007; Jia, 2008; Radi et al., 2009; Ahammad, 2013). In non-mediator based biosensor, the transfer of electron occurs between the electrode and the enzyme (Ahammad, 2013). The preparation process being very simple, the non-mediator based biosensors have been extensively used in the past. In the mediator based biosensor device, the mediator plays a key role to shuttle electron between the electrode and the enzyme (Scheme 1). Most commonly, methylene blue, ferrocene and carboxylic acid are used as mediators (Lin et al., 2000; Tian et al., 2001; Li et al., 2004; Tripathi et al., 2006; Senel et al., 2010). The mediator in the case of HRP is pointed to be important because the shuttling of electron between electrode and HRP is large as the active site of HRP is located deep in the protein sheath (Ahammad, 2013). In our current device, Os acts as a mediator where Os^2+^ is reduced to Os^3+^ in the process (Scheme 1). Different modified electrode has been introduced in the past, the detection limit in most case were in the range of μM concentration with a limited contribution where mediator-based HRP biosensor reached a lower detection limit in the range of tens-hundreds nm concentration (Chen et al., 2006; Zhang et al., 2008; Lu et al., 2010).

Using Os-HRP modified carbon electrode, we have observed a change in the reduction current during illumination of PSII membranes reflecting the production of H_2_O_2_ (Figure 4A). A change of ~400 pA reflects generation of H_2_O_2_ which was then found to be stable in the period of 20–60 min of light illumination. The reduction current was found to drop immediately under dark condition. Based on the considerable changes monitored in reduction current, the biosensor device is claimed to be sensitive for its application in photosynthetic samples.

ROS generated in the cell or organelles are eliminated by antioxidant enzymes such as SOD, glutathione peroxidase, CAT or by low molecular antioxidants such as vitamins, glutathione etc. (Halliwell and Gutteridge, 2007). When SOD which dismutates O${}_{2}^{•-}$ to H_2_O_2_ was added to the illuminated PSII membrane, the reduction current for H_2_O_2_ was observed to gradually enhance leading to the conclusion that the H_2_O_2_ generated in the PSII membrane is formed via the formation of O${}_{2}^{•-}$. The addition of SOD (Figure 4B) during the light illumination drastically enhanced the reduction current by 600 pA followed by a sudden drop indicate the fast conversion of O${}_{2}^{•-}$ into H_2_O_2_ available in the PSII pool. This is in agreement with observed result with EPR signal of the EMPO-OOH adduct observed under the effect of SOD (Figure 2A). The subsequent addition of CAT which converts H_2_O_2_ to H_2_O and molecular oxygen brings back the reduction current close to the value observed under dark condition.

Electrochemical detection of H_2_O_2_ is suggested here to be of great importance in addition to other methods that has been used in the recent past. In the detailed study performed by Šnyrychová and co-workers, different H_2_O_2_ detecting probes were tested. Probes such as amplex red and amplex ultra-red were found to be sensitive to light and thus are not appropriate for the study on the generation of H_2_O_2_ in plant sample where effect of light stress is frequently studied. Based on the results, the authors also suggested that these probes should be used with caution to avoid artifacts (Šnyrychová et al., 2009). In addition to this, the toxicity caused by the exogenous addition of probes cannot be completely excluded. The method of electrochemical measurements is also preferred because of the simplicity, low-cost, high-sensitivity and selectivity (Ahammad, 2013). The simplicity and low-cost of electrochemical measurements is because of the fact that the electrodes can be used for endless time with easy fabrication with designated mediator and enzyme and overnight incubation at 4°C under dark condition or as per standards. Based on the redox potential of the redox couple, the modified electrode are specific for different species. Os-HRP modified electrode is specific for H_2_O_2_ and thus can be widely used in photosynthetic research where detection of H_2_O_2_ has always been a challenge. The sensitivity of the modified electrode depends on the size and the material used. In our current report, Os-HRP modified carbon electrode has been claimed to be sensitive and highly selective among other H_2_O_2_ detection techniques available till date. This fact opens the possibility for using Os-HRP modified electrode for H_2_O_2_ detection in photosystem I (PSI) where H_2_O_2_ is formed by the transfer of electron from reduced ferredoxin to molecular oxygen via ferredoxin-thioredoxin reductase (Gechev et al., 2006). Plasma membrane NADPH oxidase complex are considered as the major producer of ROS including O${}_{2}^{•-}$ and H_2_O_2_ in cells (Sagi and Fluhr, 2001, 2006; Halliwell and Gutteridge, 2007). In addition to this, among the enzymatic source of O${}_{2}^{•-}$ and H_2_O_2_, cell wall bound peroxidases, aminooxidases, flavin containing oxidases, oxalate, and plasma membrane oxidases are known to be involved (Bolwell et al., 2002; Mori and Schroeder, 2004; Svedruzic et al., 2005). In the case of apoplastic oxidative burst, ROS are produced by cell wall bound oxidases, peroxidases and polyamine oxidases (Minibayeva et al., 1998, 2009). Thus, the current electrode also opens the possibility for measuring generation of H_2_O_2_ from different and localized structures of plants and animals cells.

## Author contributions

AP and SK contributed to the conception and design of the work. AP, AK, MS, HK, TS performed the measurements. AP analyzed, interpreted the data and drafted the manuscript. AK, PP participated in drafting the manuscript. SK, MK, and MT revised it critically for important content. All authors approved the final version of the manuscript.

### Conflict of interest statement

The authors declare that the research was conducted in the absence of any commercial or financial relationships that could be construed as a potential conflict of interest.
