# Supplementary figures and images for: Detection of hydrogen peroxide in Photosystem II (PSII) using catalytic amperometric biosensor
Source: Front Plant Sci. 2015 Oct 15;6:862. doi: 10.3389/fpls.2015.00862 (PMC4606053; doi:10.3389/fpls.2015.00862)

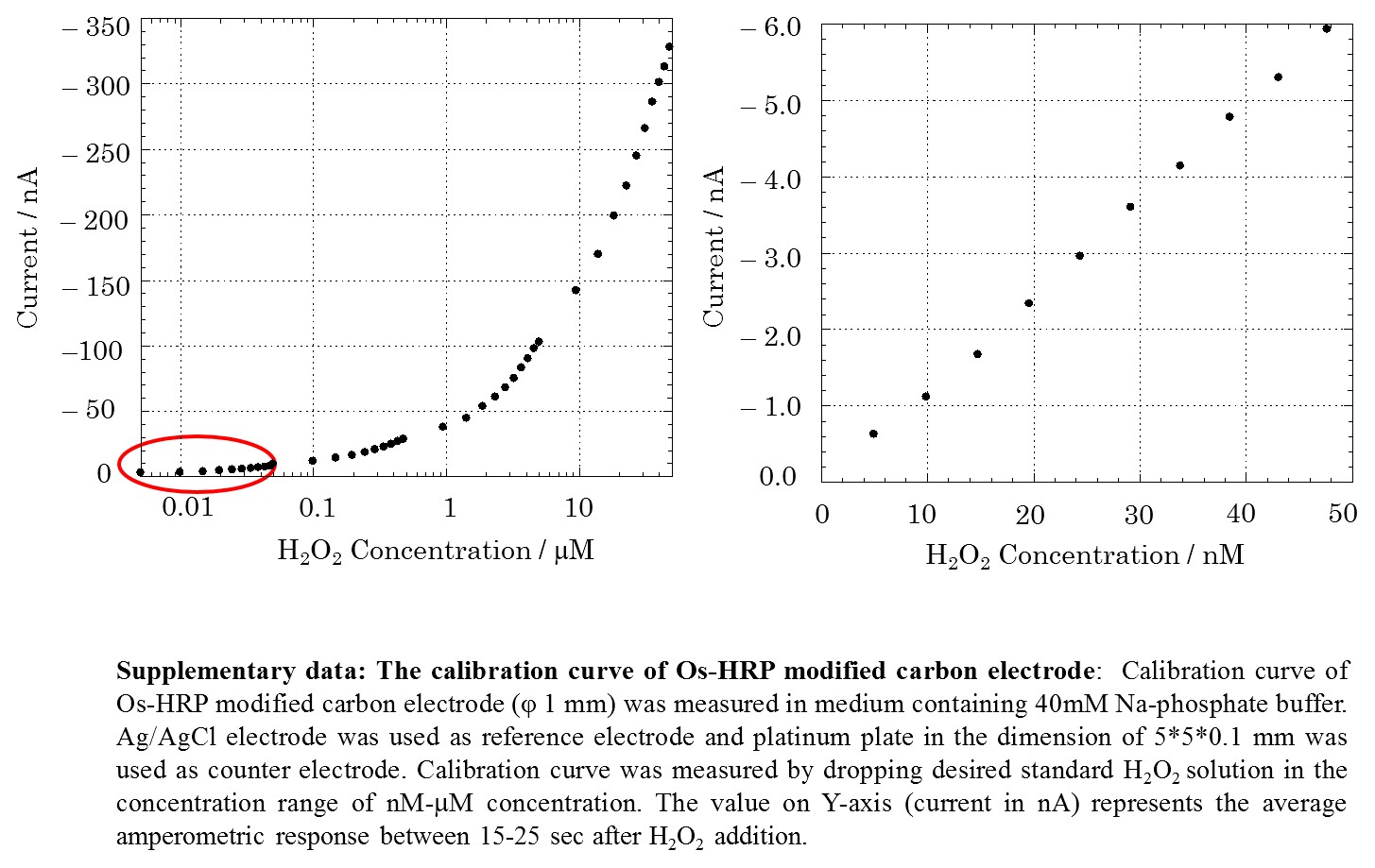

Supplement: Supplementary file 1 [file Image1.JPEG]
